# Electrocardiogram challenge: acute coronary occlusion in a ventricular paced rhythm diagnosed using Sgarbossa criteria

**DOI:** 10.1093/ehjcr/ytag166

**Published:** 2026-03-06

**Authors:** Michael Gomes

**Affiliations:** Department of Cardiology, Royal Darwin Hospital, 105 Rocklands Drive, Tiwi, NT 0810, Australia

## Clinical vignette

**Figure 1:**
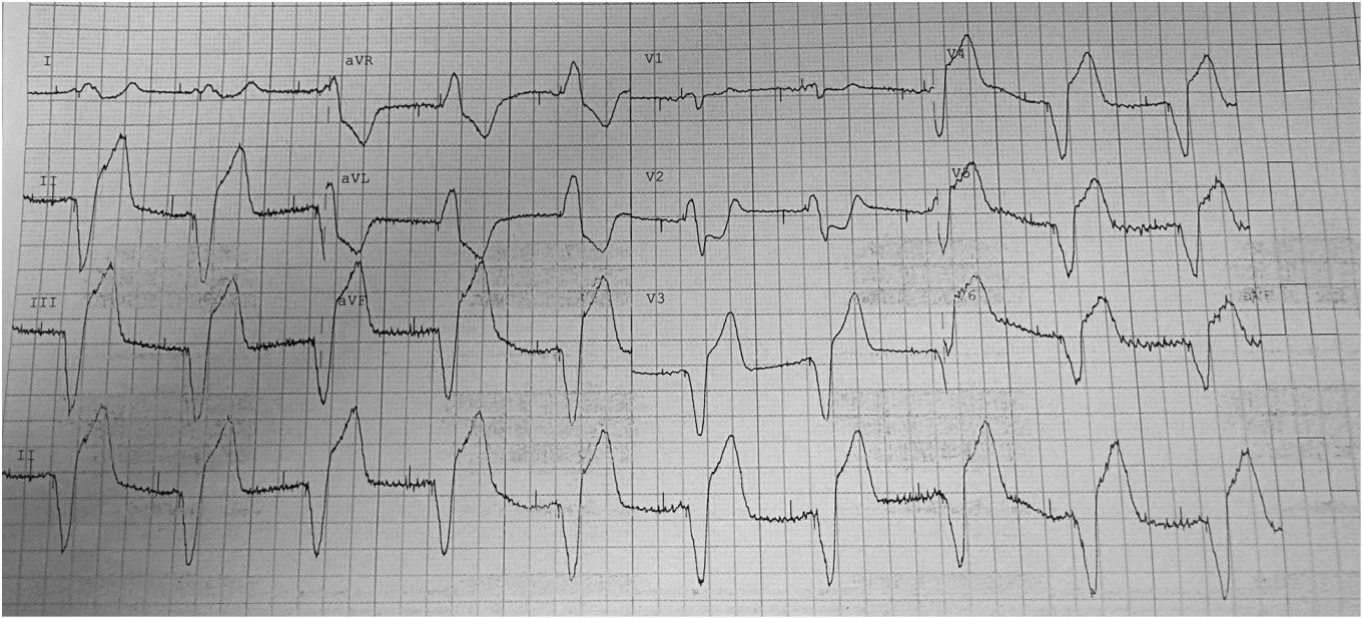
Twelve-lead ECG at presentation demonstrating a ventricular paced rhythm with concordant and excessively discordant ST-segment changes fulfilling Sgarbossa and modified Sgarbossa criteria, consistent with acute coronary occlusion.

A 76-year-old man with a permanent pacemaker implanted for high-grade atrioventricular block presented with acute chest pain, diaphoresis, and hypotension. He had multiple cardiovascular risk factors and no prior history of coronary revascularization. On arrival, he remained haemodynamically unstable with ongoing chest discomfort. The 12-lead electrocardiogram (ECG) demonstrated a ventricular paced rhythm with marked ST-segment abnormalities, including ST depression in the anterior leads and prominent ST elevation in the inferior leads *Figure 1*. These ST-segment changes appeared disproportionate to the paced QRS morphology. Given concern for acute coronary occlusion, urgent cardiology consultation was obtained and coronary angiography was being arranged. Before transfer to the catheterization laboratory, the patient developed ventricular tachycardia arrest requiring defibrillation, with return of spontaneous circulation achieved. Emergent coronary angiography demonstrated an acutely occluded proximal right coronary artery, which was treated successfully with primary percutaneous coronary intervention and drug-eluting stent implantation, restoring Thrombolysis In Myocardial Infarction Grade 3 (TIMI 3) flow.

## Multiple-choice questions

### Question 1

Which original Sgarbossa criteria are fulfilled on this ECG?

- Concordant ST elevation ≥1 mm
- Concordant ST depression ≥1 mm in V1–V3
- Discordant ST elevation ≥5 mm
- Both B and C
- A, B and C

**Correct answer: D**

## Explanation

This ECG demonstrates concordant ST depression ≥1 mm in lead V2, fulfilling the second original Sgarbossa criterion. In addition, there is excessive discordant ST elevation ≥5 mm in the inferior leads with predominantly negative QRS complexes, fulfilling the third original criterion. Concordant ST elevation ≥1 mm is not present. The presence of two original Sgarbossa criteria significantly increases the likelihood of acute coronary occlusion in ventricular paced rhythms.^1–3^

### Question 2

Which ECG feature most strongly supports acute coronary occlusion in a ventricular paced rhythm?

- Wide QRS duration
- Discordant ST elevation <1 mm
- Concordant ST elevation ≥1 mm
- T-wave inversion in lateral leads
- Prolonged PR interval

**Correct answer: C**

## Explanation

Concordant ST elevation ≥1 mm in a lead with a predominantly positive QRS complex fulfils the first original Sgarbossa criterion and carries the highest diagnostic weight in the original scoring system. In this ECG, concordant ST elevation is not present. Although V2 demonstrates ST depression consistent with posterior infarction physiology (a mirror image of posterior ST elevation), this represents concordant ST depression rather than concordant ST elevation. Recognition that concordant ST elevation is absent is important when applying the original criteria accurately.^1–3^

### Question 3

What is the principal modification introduced in the modified Sgarbossa criteria?

- Removal of concordant ST depression from the scoring system
- Replacement of absolute discordant ST elevation ≥5 mm with a proportional ST/S ratio ≥0.25
- Addition of QRS duration measurement
- Inclusion of reciprocal T-wave inversion
- Requirement for biomarker elevation before ECG interpretation

**Correct answer: B**

## Explanation

The modified Sgarbossa criteria replace the absolute threshold of ≥5 mm discordant ST elevation with a proportional rule based on the ST-segment elevation to S-wave amplitude ratio (≥0.25). This modification improves sensitivity while maintaining specificity for detecting acute coronary occlusion in left bundle branch block and paced rhythms. It accounts for expected repolarization abnormalities relative to QRS amplitude rather than using a fixed millimetre cutoff.^1–4^

## Supplementary Material

ytag166_Supplementary_Data

## Data Availability

No new data were generated or analysed in support of this research.

## Supplementary material

**Consent:** Written informed consent was obtained from the patient for publication of this case and accompanying images.
