# Supplementary material for: Electrocardiogram challenge: acute coronary occlusion in a ventricular paced rhythm diagnosed using Sgarbossa criteria
Source: Eur Heart J Case Rep. 2026 Mar 6;10(3):ytag166. doi: 10.1093/ehjcr/ytag166 (PMC12998530; doi:10.1093/ehjcr/ytag166)
Supplement: ytag166_Supplementary_Data [file ytag166_supplementary_data.zip › Supplementary FIgure Legends.docx]

## Supplementary Material

Supplementary Figure S1: Coronary angiography demonstrating proximal RCA occlusion.

Supplementary Figure S2: Final angiographic result after PCI with restoration of TIMI 3 flow.
