# Supplementary figures and images for: Electrocardiogram challenge: acute coronary occlusion in a ventricular paced rhythm diagnosed using Sgarbossa criteria
Source: Eur Heart J Case Rep. 2026 Mar 6;10(3):ytag166. doi: 10.1093/ehjcr/ytag166 (PMC12998530; doi:10.1093/ehjcr/ytag166)

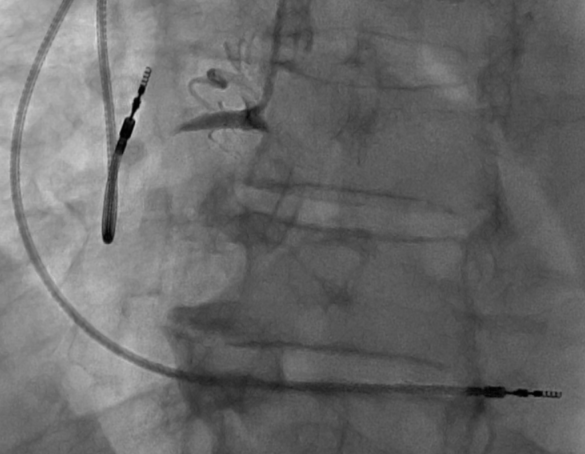

Supplement: ytag166_Supplementary_Data [file ytag166_supplementary_data.zip › angio.png]

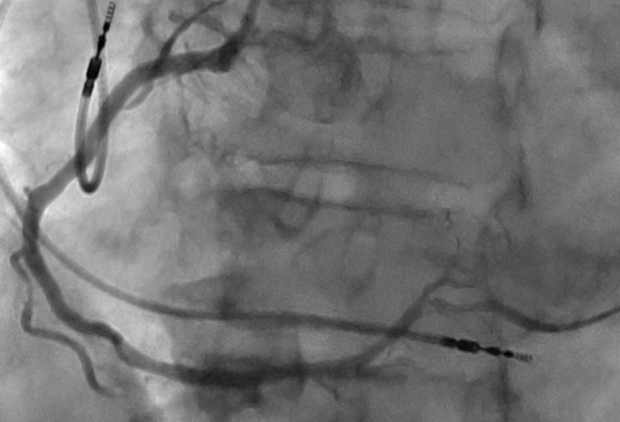

Supplement: ytag166_Supplementary_Data [file ytag166_supplementary_data.zip › angiopostpci.png]
